# A robust pooled testing approach to expand COVID-19 screening capacity

**DOI:** 10.1371/journal.pone.0246285

**Published:** 2021-02-08

**Authors:** Douglas R. Bish, Ebru K. Bish, Hussein El-Hajj, Hrayer Aprahamian

**Affiliations:** 1 University of Alabama, Information Systems, Statistics, and Management Science, Blacksburg, VA, United States of America; 2 Virginia Tech, Industrial and Systems Engineering, Blacksburg, VA, United States of America; 3 Industrial and Systems Engineering, Texas A&M University, College Station, TX, United States of America; West Virginia University, UNITED STATES

## Abstract

Limited testing capacity for COVID-19 has hampered the pandemic response. *Pooling* is a testing method wherein samples from specimens (e.g., swabs) from multiple subjects are combined into a pool and screened with a single test. If the pool tests positive, then new samples from the collected specimens are individually tested, while if the pool tests negative, the subjects are classified as negative for the disease. Pooling can substantially expand COVID-19 testing capacity and throughput, without requiring additional resources. We develop a mathematical model to determine the best *pool size* for different *risk groups*, based on each group’s estimated COVID-19 prevalence. Our approach takes into consideration the sensitivity and specificity of the test, and a dynamic and uncertain prevalence, and provides a *robust* pool size for each group. For practical relevance, we also develop a companion COVID-19 pooling design tool (through a spread sheet). To demonstrate the potential value of pooling, we study COVID-19 screening using testing data from Iceland for the period, February-28-2020 to June-14-2020, for subjects stratified into high- and low-risk groups. We implement the robust pooling strategy within a *sequential* framework, which updates pool sizes each week, for each risk group, based on prior week’s testing data. Robust pooling reduces the number of tests, over individual testing, by 88.5% to 90.2%, and 54.2% to 61.9%, respectively, for the low-risk and high-risk groups (based on test sensitivity values in the range [0.71, 0.98] as reported in the literature). This results in much shorter times, on average, to get the test results compared to individual testing (due to the higher testing throughput), and also allows for expanded screening to cover more individuals. Thus, robust pooling can potentially be a valuable strategy for COVID-19 screening.

## Introduction

With around 46.8 million confirmed cases and 1.2M deaths in at least 188 countries (as of 11/2/2020) [1], the COVID-19 pandemic continues to be the cause of considerable suffering and economic disruption. Effective mitigation requires laboratory-based testing to identify COVID-19 positive subjects. As the WHO Director-General Ghebreyesus puts it, "The most effective way to prevent infections and save lives is breaking the chains of transmission, and to do that you must test and isolate. We cannot stop this pandemic if we don’t know who is infected. We have a simple message for all countries: test, test, test” [2]. Testing is essential not only for symptomatic individuals, but also for asymptomatic individuals [3], because they are a major source of transmission [4].

Unfortunately, a major impediment to mitigation efforts in the United States (US), and in other parts of the world, has been the limited testing capacity for COVID-19, which, in the US, has constrained the number of tests that could be conducted, mostly restricting testing to those with symptoms for case identification, and less on mass screening and contact tracing efforts. Countries that were able to ramp up their COVID-19 testing capacity quickly and follow an aggressive testing strategy earlier during the epidemic were considerably more effective than others. For example, South Korea was able to curb the growth of the disease mainly through expanded testing, without a strong imposition of social distancing measures. By testing over 300,000 people out of its population of 52 million [5], South Korea was able to implement highly effective interventions early on, including contact tracing, followed by enforced quarantines and isolations. Another example is Iceland, which is able to offer free COVID-19 testing to the general population [6], and as of June 14, 2020, was able to test around 17% of its population [7].

The current mode of COVID-19 testing worldwide is *individual testing*, that is, each subject’s specimen is tested with a single test. An alternative to individual testing, proposed by Dorfman in 1943 [8], is *pooled testing*, in which samples, sufficient for testing, are extracted from the specimens (e.g., nasopharyngeal swabs) from multiple subjects, and combined in a *pool*, and tested via a single test; if the pooled test’s outcome is positive, then all subjects in the pool are individually tested (with the same type of test, using a new sample extracted from the previously collected specimen); and if the pooled test’s outcome is negative, then all subjects in the pool are classified as disease-negative. Pooled testing does not require any additional resources beyond individual testing [9], and can substantially expand testing capacity over individual testing, especially when prevalence rates are low. Pooled testing, and in particular the Dorfman pooling method, is currently used in public health screening, including screening for sexually-transmitted diseases and screening donated blood for transfusion-transmittable infections [10–13]. An important design decision in pooled testing is the *pool size* (i.e., number of specimens in each pool) so as to maximize the efficiency of testing (i.e., minimize the number of tests per subject) [11] provides a rigorous analysis on the selection of an optimal pool size for Dorfman pooling. Because pooling has obvious application to COVID-19 testing, there have been several recent pooling papers focused on COVID-19, for example [14–16], offer various simplifications of the analysis in [11], with a focus on COVID-19. Unlike [14–16], this paper considers uncertainty in the prevalence rate, which is a defining feature of COVID-19; in other public health screening applications, the disease spread is less dynamic and prevalence changes at a much slower rate (e.g., sexually-transmitted diseases). For COVID-19, disease prevalence changes quickly, and there is high uncertainty around its prevalence at any point in time.

Considering pooled testing design, one concern is accuracy, in particular a potential increase in the number of false-negatives as a result of pooling. Pooling does not increase the number of false-positives over individual testing [11]. With imperfect tests, pooling will increase the false-negative rate (e.g., see [11]), because a disease-positive subject must be tested twice (first in a pool, then individually) to be classified as positive. Further, pooling may lead to the “dilution” of the infected specimen(s) in the pool, reducing the sensitivity of the pooled test, thus increasing the false-negative rate, especially for larger pools [13]. A common method for reducing dilution is to set a maximum pool size for, e.g., see [10, 12].

There are several lab-based studies that investigate the impact of pooling on the sensitivity of a PCR test for COVID-19, which is the most common type of test for COVID-19 screening. This preliminary research indicates that pooled testing leads only to a minor reduction in PCR sensitivity for larger pool sizes. For example, [9] shows that the PCR test was able to detect an infected specimen in pools of size of 32 in nine out of the ten pools tested. [17] shows a similar result for pools of size 30. In addition, several other studies find no loss of sensitivity for smaller pools, e.g., [18–20] use pool sizes of 5, 8, and 10, respectively. Not surprisingly [21, 22], show that the dilution effect is more pronounced when the infected specimens in the pool have low viral loads; the window period analysis in [21] indicates that the low viral load typically occurs for specimens that are collected either too early or too late after infection. Related to the overall pooled testing design [23], proposes a design where the pooled test is repeated to reduce false-negatives, and shows that such a design is still efficient compared to individual testing. On the other hand [24], explores, via simulation, different pooling designs (e.g., adaptive and non-adaptive) and different metrics (e.g., efficiency and accuracy).

A PCR test run takes in the order of 2 hours [25, 26] to complete, thus, pooled testing, followed by individual testing as needed, is viable from a time perspective, and does not significantly increase the testing time for a particular subject. Further, testing machines have a capacity, which limits their throughput (e.g., number of completed tests per testing day), e.g., a limit of 96 samples per testing cycle (run) is common [25]. Because pooled testing increases the number of specimens tested per testing cycle, it also increases the throughput, shortening the average time to get the test results. The expanded testing capacity provided by pooling could enable more extensive testing for COVID-19.

This paper builds on previously published mathematical models by the authors; develops a robust approach for pooled testing design that can be customized for different testing populations and for a dynamic and uncertain disease prevalence; and demonstrates that the proposed pooled testing approach has the potential to substantially increase COVID-19 testing capacity. Further, we explore the impact of test sensitivity, and provide insight on how to manage the uncertainty in test sensitivity. This research is timely as limited COVID-19 testing capacity remains a serious problem, and more testing capacity is urgently needed, especially given the increasing number of infections, and the efforts to return to some form of “normalcy.”

## Methods

We study case identification via pooled testing; develop an easily implementable method for robust pooling design under prevalence uncertainty [27, 28], and for risk-stratified groups; and illustrate the benefits through a case study on mass screening for COVID-19. Our model builds on our earlier work, in particular, we take the analytically complex models and results from [10], which uses risk-based pooling (to determine pool sizes and pool assignments for subjects given their individual disease risk), from [11], which uses robust optimization (to determine pool sizes under prevalence uncertainty), and from [29], which develops sequential pooling design for surveillance; and we develop a novel method for designing a simple, *robust pooling* strategy, which can be incorporated into a *sequential pooling design* framework to consider infection dynamics and prevalence uncertainty. For practical relevance, we develop a companion COVID-19 pooling design tool (through a spread sheet), which will be available online.

We consider a PCR test, which can be used for both individual and pooled testing. For pooled testing, we consider the Dorfman method [8] (hereafter, “pooling”). The test produces a binary outcome, with a positive (negative) outcome indicating the presence (absence) of the infection in the pool (for pooled testing) or in the individual specimen (for individual testing). We let *Se* and *Sp* respectively denote the test *sensitivity* (true positive probability) and *specificity* (true negative probability), and assume that pooling does not change the test’s efficacy up to a *maximum allowable pool size*. The terms “subject” and “specimen” respectively refer to the individual to be tested, and specimen collected from the individual, and are used interchangeably. We assume that each specimen has sufficient material (samples) for multiple tests, thus, if an individual follow-up test is needed for a subject, it is conducted on a new sample extracted from the subject’s previously collected specimen.

### Model

We design a pooling strategy for a testing population divided into *risk groups* based on each group’s estimated disease prevalence, by determining, for each group, a *robust pool size*. The objective is to maximize the efficiency of testing (minimize the expected number of tests per subject) under unknown prevalence. In the following, we detail the derivation of the robust pool size, discuss its extension to sequential pooling design, and derive the false-negative rate of pooling.

To simplify the subsequent notation, we omit the group index. Consider a given group, and let *P* denote the unknown prevalence for the group, with uncertainty set (range) *S(P) =* [*L*, *U*], which consists of discrete *prevalence scenarios*, each with probability, *Pr(P = p)*, *p ϵ S(P)*; our method extends to any user-specified discrete or continuous distribution for *P*. The uncertainty set *S(P)* is estimated by the tester, and does not necessarily correspond to the true support of random variable *P*, which is unknown in practice.

### Testing efficiency

Let *n*_*max*_ denote the maximum allowable pool size (due to technological limitation, dilution effect, etc.). If a pool with *n* specimens tests negative, then only *1/n* tests are needed per subject; and if the pool tests positive, then each subject requires an individual “follow-up” test, leading to *1+1/n* total tests per subject (Table 1 displays the probability of each possible event). Then, for any pool size *n* and prevalence scenario *p*, the expected number of tests per subject tested (“expected tests”) under pooling, denoted by E[*T*(*n*,*p*)], follows:

$$E[T(n,p)]=\frac{1+n[Se-(Se+Sp-1){\left(1-p\right)}^{n}]}{n}.$$ (1)

**Table 1:** Probabilities of all possible events in pooled testing (for a Pool of *n* Subjects).

Pooled Test Outcome True Subject Status in Pool	Pooled Test Negative	Pooled Test Positive
	(Pooled Test Only)	(Pooled Test Plus *n* Individual Tests)
All Subjects Negative	*Sp*(1−*p*)^*n*^	(1−*Sp*)(1−*p*)^*n*^
At Least One Subject Positive	(1−*Se*)(1−(1−*p*)^*n*^	*Se*(1−(1−*p*)^*n*^)

The probabilities for the intersections of all possible pooled test outcomes and subject status in pooled testing.

A key characteristic of COVID-19 is dynamic and uncertain prevalence rates, thus during testing the actual prevalence rate is unknown. To develop a pool size that is robust under prevalence uncertainty, we use the *Regret* measure (e.g., [11]), which can be computed for any pool size *n* and prevalence scenario *p* as follows:

Regret(n,p)=E[T(n,p)]−E[T(n(p),p)], (2)

where *n*(*p*) is the pool size that minimizes E[T(*n*,*p*)] for prevalence scenario *p (i*.*e*., the minimizer of Eq (1) under *perfect information* on *p*, the prevalence rate), and can be approximated as follows [11]:

$$n(p)\approx {argmin}_{\left(n\in (\lfloor \tilde{n}(p)\rfloor ,\lceil \tilde{n}(p)\rceil ,n_{max}\right)}[E[T(n,p)]],$$ (3)

where $\tilde{n}(p)=\sqrt{\frac{1}{p(Se+Sp-1)}}$.

In Eq (3), we evaluate the expected tests, E[T(*n*,*p*)], at the ceiling and floor of $\tilde{n}(p)$, and at the maximum allowable pool size, *n*_*max*_, and select the value that yields a lower expected number of tests, in order to obtain an integer pool size, *n*(*p*). A complex algorithm that derives the (exact) optimal pool size for scenario *p* is provided in our previous work [11].

The perfect information assumption used in Eq (3), which implies a known prevalence rate, is obviously not realistic, and is utilized for the purpose of deriving a robust pool size when the prevalence rate is uncertain. Specifically, we determine a *robust pool size*, n*, so as to minimize the expected *Regret* over the uncertainty set *S(P)* of random variable *P*, that is:

$$n^{*}={argmin}_{\left(n\in Z^{+},n\leq n_{max}\right)}[\sum_{p\in S(P)}Pr(P=p)\times Regret(n,p)].$$ (4)

Utilizing the *Regret* objective in Eq (4) requires the determination of a set of perfect information pool sizes *a priori* via Eq (3). The *Regret* objective leads to a robust solution that is not overly conservative, and a simple method for determining a robust pool size, compared to the analytically complex algorithm of [11].

In summary, to design a robust pooling strategy under prevalence uncertainty, we use Eqs (3) and (4) with each risk group’s respective parameters. Specifically, using Eq (3), we first calculate a set of perfect information *pool sizes*, *n*(*p*), for each scenario *p* in the range of *P*; and then use Eq (4) to determine the robust pool size for the group under prevalence uncertainty, n*. This process is automated in our spread sheet, and the user needs to only input the problem parameters, including any user-specified probability distribution (discrete or continuous) for random variable *P*.

### Sequential pooling design

Due to infection dynamics, we allow for updates to pooling design in each testing period (see, e.g., [29] for sequential pooling design for surveillance). To present our framework for sequential pooling design, we use index *t* to denote testing period *t*∈*Z*^+^. At the beginning of each testing period *t*, we update the uncertainty set of random variable *P* based on the testing data obtained in periods 1,*…*, *t -1*, compute each group’s pool size in period *t* (Eqs (3) and (4)), and use the updated pool sizes in testing period *t*. We repeat this process through the testing horizon.

### False-negative rate

We determine the expected false-negatives (*FN*s) per subject tested under pooling. When a pool contains infected specimen(s), *FN*(s) occurs if the pool tests negative, or the pool tests positive but the individual follow-up test for an infected specimen is negative, leading to:

$$E[{FN}^{Pool}]=(1-{Se}^{2})p.$$ (5)

On the other hand, for individual testing, when a subject is infected (with probability *p*), the test falsely provides a negative outcome (with probability 1-*Se*), leading to:

$$E[{FN}^{Individual}]=(1-Se)p.$$ (6)

### Data

We demonstrate the efficiency of robust pooling for mass screening using published data for COVID-19, which is stratified into low- and high-risk groups based on symptom class. To this end, we construct each group’s uncertainty set for the prevalence random variable to correspond to the 95% confidence interval (CI) from the dataset, with 100 equally-spaced prevalence scenarios (each with equal probability) within each uncertainty set; and develop a robust pooling design for each group (via Eqs (3) and (4)).

We consider the PCR test for COVID-19 testing. In [9], ten pools of size 32 were constructed, where each pool contained one specimen infected with COVID-19 and 31 infection-free specimens. Using the PCR test, nine pools tested positive and one pool tested negative (due to dilution). Other studies suggest that the sensitivity of the PCR test for COVID-19 can be as low as 0.71 and as high as 0.98 [30]. As a result, we perform a one-way sensitivity analysis on the test sensitivity parameter, *Se*, over a wider range, of [0.70, 1.00], discuss the specific results for the published values (i.e., *Se* values of 0.71 and 0.98) in detail, and qualitatively discuss the results for other test sensitivity values. We also assume that the test has perfect specificity, i.e., *Sp = 1* [31], which is not subject to the dilution effect, and *n*_*max*_ = 32 [9].

#### Case study data

The case study focuses on mass screening of high- and low-risk subjects based on Iceland’s COVID-19 testing dataset [7]. This dataset includes 63,134 subjects, and reports the number of subjects screened and the number of positive test outcomes for COVID-19 per day, based on testing data from two laboratories, which collectively conduct all COVID-19 screening in Iceland: 1) 21,576 high-risk subjects with “severe symptoms and/or are at high risk of infection because of close contact with a diagnosed individual” [32], tested by the National University Hospital of Iceland (NUHI) between February-28-2020 and June-14-2020, leading to 1,628 positive-testing subjects; and 2) 41,558 low-risk subjects in the general population who have requested screening on a voluntary basis, tested by deCODE genetics between March-15-2020 and June-14-2020, leading to 182 positive-testing subjects.

We implement sequential pooling design, and compute the robust pool sizes for every week between March-05-2020 and June-14-2020 (high-risk group), and between March-19-2020 and June-14-2020 (low-risk group), i.e., after obtaining a number of days of testing data for each group. In particular, at the beginning of each week, we use the previous week’s testing data for each of the low- and high-risk groups, and the Wald’s method [33], to construct a 95% CI for each group’s prevalence:

$$L(\hat{p})=\hat{p}-z_{0.975}\sqrt{\hat{p}(1-\hat{p})/T},\;\mathrm{and}\;U(\hat{p})=\hat{p}+z_{0.975}\sqrt{\hat{p}(1-\hat{p})/T},$$

where $\hat{p}$ is the point estimate, *z*_0.975_ is the inverse of the CDF of the standard normal distribution at point 0.975, and T is the number of subjects tested in the previous week.

## Results

**Fig 1:**
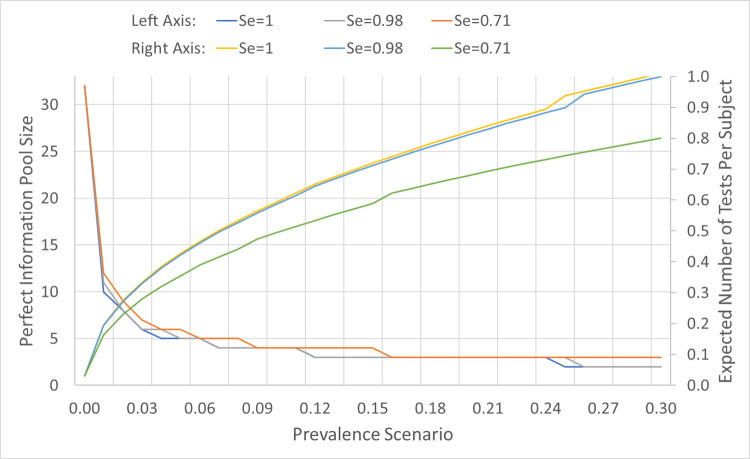
Perfect information pool size and expected number of tests per subject versus prevalence rate for various test sensitivity values. The perfect information pool size, *n(p)*, and expected number of tests per subject, *E[T(n(p)*,*p)]*, for each prevalence scenario *p* between 0.01–0.292 for test sensitivity values, *Se* = 0.71, 0.98, 1.

**Fig 2:**
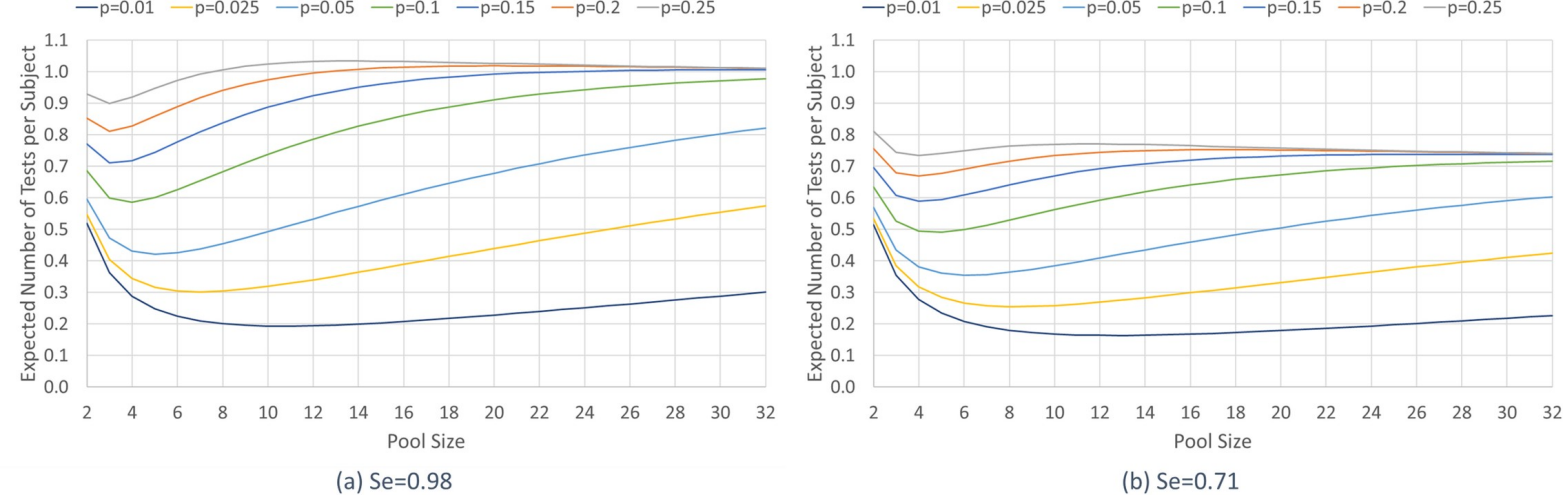
Expected number of tests per subject versus pool size for various prevalence rates for test sensitivity values (a) *Se* = 0.98 and (b) *Se* = 0.71. The expected number of tests per subject, *E[T(n(p)*,*p)]*, for various prevalence scenarios *p* between 0.01–0.25 for test sensitivity values (a) *Se* = 0.98 and (b) *Se* = 0.71 for pool sizes from 2–32.

For illustrative purposes, Fig 1 reports the perfect information pool sizes (i.e., (*n(p)*) from Eq (3) for prevalence scenario *p*) and expected number of tests per subject (simply, expected tests), for various prevalence scenarios between 0.01 and 0.292, and test sensitivity values (*Se* = 0.71, 0.98, 1), considering that the test has perfect specificity. The expected tests under perfect information on prevalence rate provides a *lower bound* (*LB*) on the number of tests achievable via pooling, but is not realistic due to the inherent uncertainty in the prevalence rate (i.e., the lack of perfect information), which is typically the case for a highly contagious disease like COVID-19. Pooling is not more efficient than individual testing for prevalence scenarios above 0.292, due to the large number of individual follow-up tests. Relatedly, Fig 2 shows the expected tests (*E[T(n(p)*,*p)]*) for various prevalence scenarios between 0.025 and 0.30, and test sensitivity values of 0.98 (Fig 2(A)) and 0.71 (Fig 2(B)) over a range of pool sizes, and thus shows the impact of pool size on efficiency for each prevalence scenario and test sensitivity.

Based on the Iceland dataset [7], we develop a robust pooling design within a sequential framework for the high-risk (those tested by NUHI) and low-risk (those tested by deCODE) groups, updated every week during the study period. We consider that the weekly robust pool sizes are used each day of that week, and examine the daily testing results.

**Fig 3:**
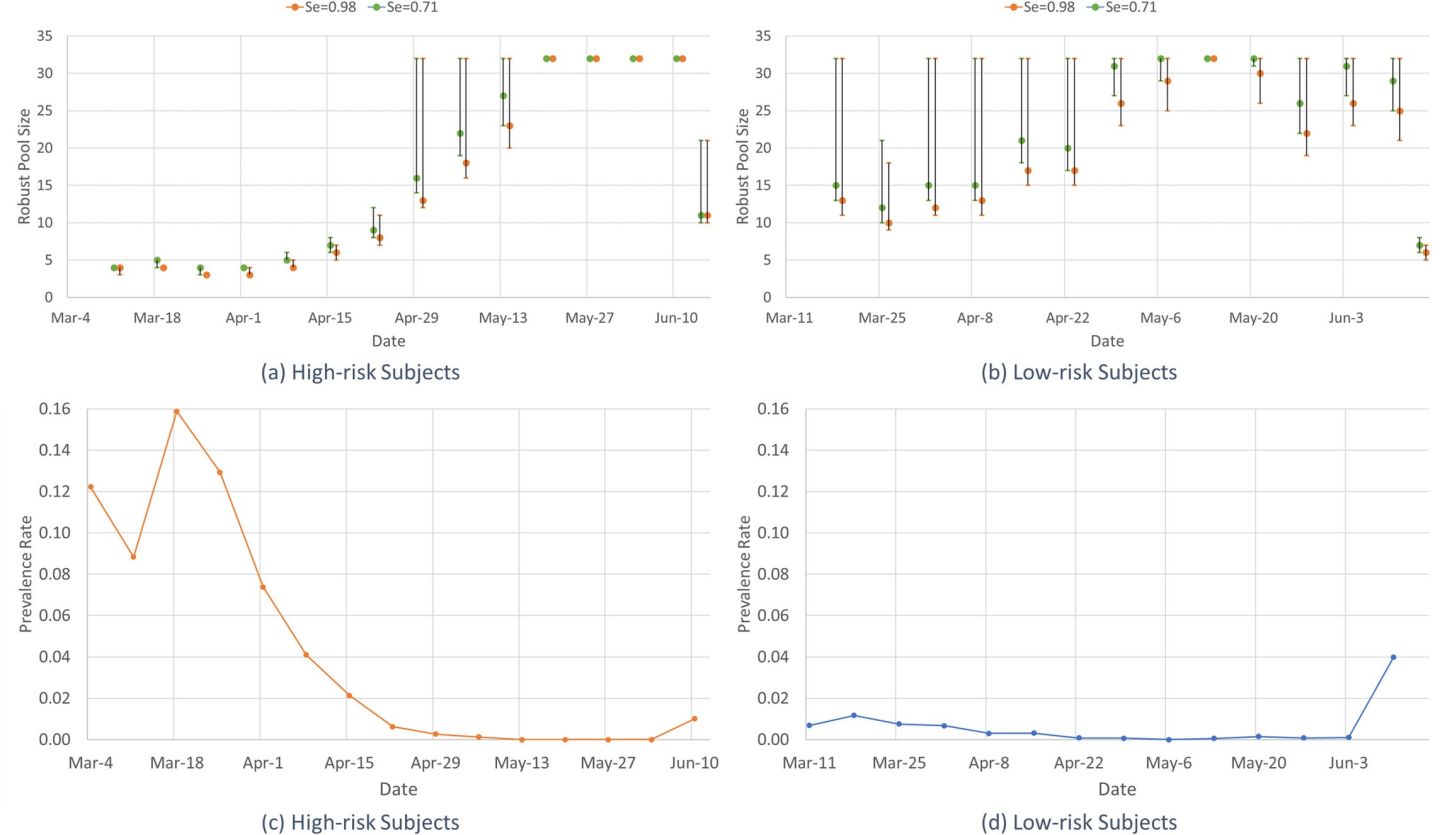
Weekly robust pool size and 95% confidence interval pool sizes for (a) High-risk and (b) Low-risk groups for two test sensitivity values, and actual weekly prevalence rates for (c) High-risk and (d) Low-risk groups. The robust pool size, n*, for each week along with pool sizes corresponding to the 95% confidence interval of the prevalence forecast for (a) high-risk and (b) low-risk groups for test sensitivity values, *Se* = 0.71 and 0.98, and the actual weekly prevalence rates for (c) high-risk and (d) low-risk groups.

Fig 3 depicts the weekly robust pool sizes (n*) for two test sensitivity values (*Se* = 0.71, 0.98) as well as the optimal pool size for each prevalence scenario (i.e., the perfect information pool size for each *p*, *n(p)*, from Eq (3)) within the 95% CI on the weekly prevalence forecast (with each CI discretized into 100 equally-spaced prevalence scenarios), along with the actual weekly prevalence rate. Note that *n(p)* decreases as the prevalence rate *p* increases (Eq (3)). Thus, the robust pool size (n*) is bounded from above by the optimal pool size for the lower limit of the CI on prevalence, and from below by the optimal pool size for the upper limit of the CI. We see that the robust pool size tends to be closer to the optimal pool size for the upper limit of the CI, that is, closer to the lower bound pool size. This figure also shows that as the test sensitivity increases, the robust pool size decreases.

**Fig 4:**
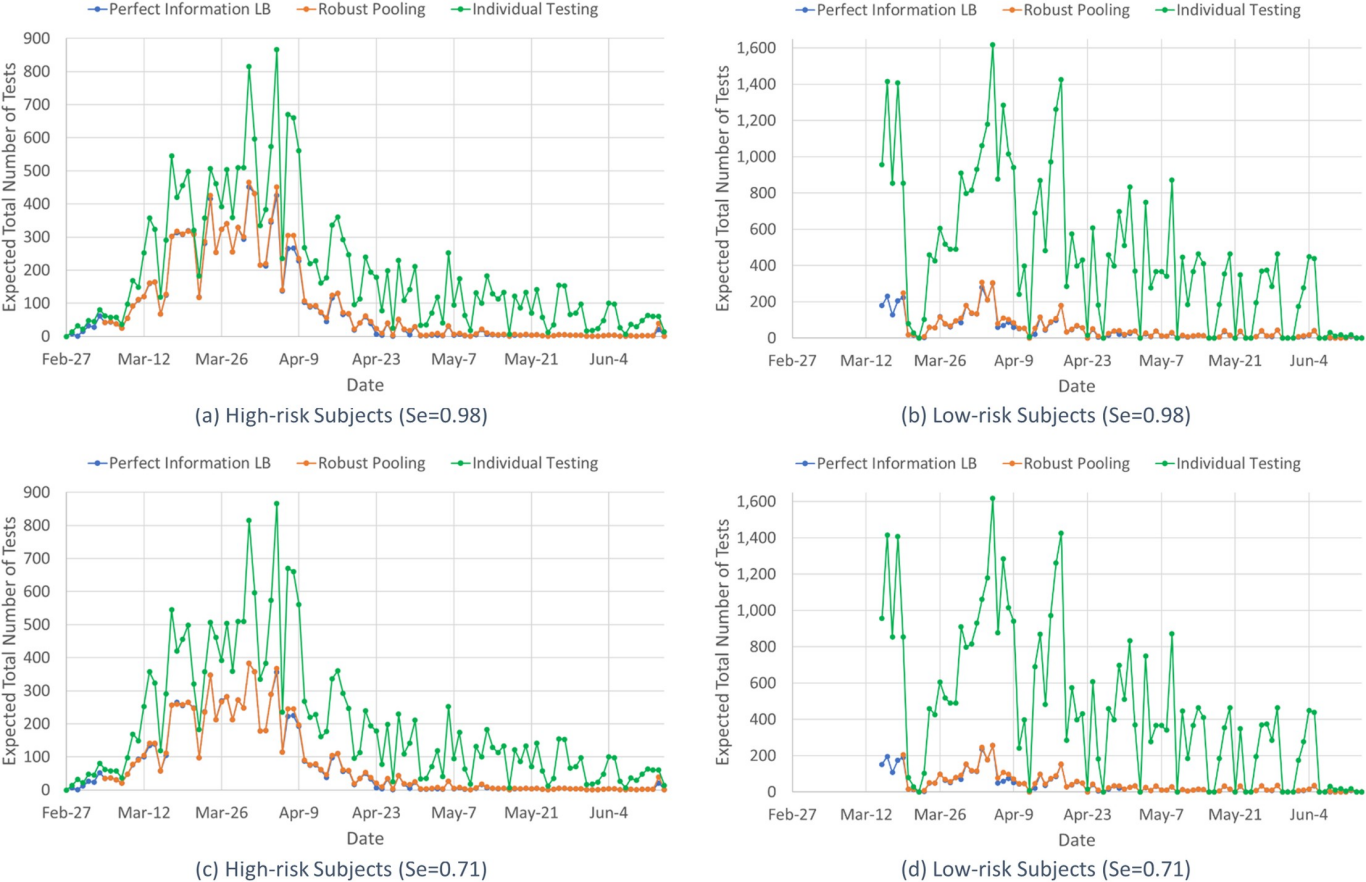
Daily expected number of tests for the high-risk group for test sensitivity values of (a) 0.98 and (c) 0.71 and for the low-risk group for test sensitivity values of (b) 0.98 and (d) 0.71. The daily expected number of tests required for the (a) high-risk group with *Se* = 0.98, (b) low-risk group with *Se* = 0.98, (c) high-risk group with *Se* = 0.71, and (d) low-risk group with *Se* = 0.71 for the perfect information lower bound, robust pooling, and individual testing.

Fig 4 reports the daily number of tests for individual testing (i.e., the actual number of tests conducted per day in the dataset), and the expected number of tests for robust pooling (i.e., using n*) and the perfect information lower bound (*LB*, i.e., using *n(p)*) for (a) high-risk and (b) low-risk subjects for a test sensitivity of 0.98, and (c) high-risk and (d) low-risk subjects for a test sensitivity of 0.71. Recall that the perfect information lower bound is unattainable, because the prevalence rate is uncertain, and a prevalence rate forecast is required due to changing disease dynamics. Considering test sensitivity values of 0.71 and 0.98, the total reduction in the number of tests, over individual testing, is 61.9% and 54.2% respectively for robust pooling (62.7% and 55.6% respectively under perfect information) for the high-risk group during the period of March-05-June-14; and 90.2% and 88.5% respectively for robust pooling (91.0% and 89.5% respectively under perfect information) for the low-risk group during the period of March-19–June-14.

In the above analysis, we use a maximum allowable pool size, *n*_*max*_, of 32, to limit the dilution effect, which is set based on preliminary studies (see Introduction), hence we next study how the value of *n*_*max*_ affects the results. Table 2 reports the efficiency of robust pooling, that is, the percent reduction in the number of tests that can be achieved via robust pooling compared to individual testing, for lower values of *n*_*max*_, and for test sensitivity values of *Se* = 0.71 and 0.98, and the high- and low-risk groups. As expected, very low *n*_*max*_ values impact the efficiency of testing more for the low-risk group compared to the high-risk group, because the latter group already uses small pool sizes due to their high prevalence.

**Table 2:** The percent reduction in the expected number of tests via robust pooling compared to individual testing for high-risk and low-risk groups and two test sensitivity values for various maximum allowable pool sizes, *n*_*max*_.

Maximum Allowable Pool Size (*n*_*max*_)	High-risk Group		Low-risk Group	
	*Se* = 0.98	*Se* = 0.71	*Se* = 0.98	*Se* = 0.71
32	54.2%	61.9%	88.5%	90.2%
30	54.1%	61.8%	88.5%	90.1%
20	54.0%	61.7%	88.2%	89.8%
10	53.4%	61%	86.2%	87.4%
5	51.3%	58.8%	79.8%	80.8%

**Fig 5:**
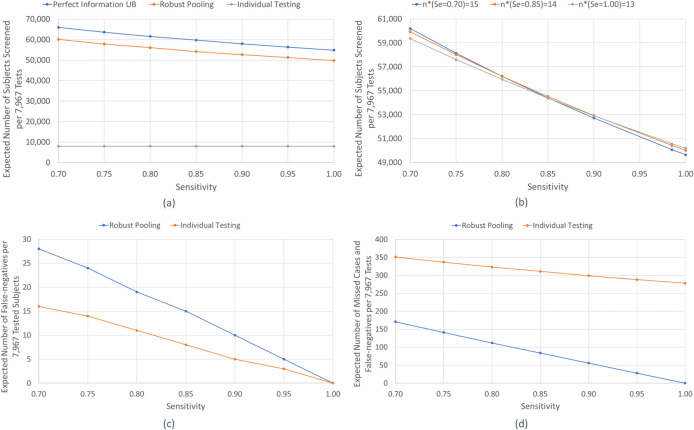
Expected number of low-risk subjects screened with 7,967 tests (a) under different strategies and (b) under different assumed test sensitivities, versus true test sensitivity, and the expected number of (c) False-negative cases and (d) Missed and False-negative Cases versus Test Sensitivity. The expected number of low-risk subjects screened with 7,967 tests (a) for the perfect information upper bound, robust pooling, n*, and individual testing versus test sensitivity, (b) assuming a prevalence rate, *p*, of 0.0065 and a test sensitivity, *Se*, of 0.70, 0.85, and 1 versus true test sensitivity, (c) *FN*s (out of the 7,967 low-risk subjects tested under both strategies), and (d) missed cases plus *FN*s for robust pooling and individual testing (out of 52,173 subjects, which corresponds to the expected number of low-risk subjects tested via robust pooling) versus test sensitivity.

By requiring, on average, fewer tests per subject, robust pooling can substantially expand testing capacity. To illustrate this concept, we select a week during the study period, i.e., the week spanning April-2 to April-8 of 2020, during which 7,967 low-risk subjects were tested via 7,967 tests, of which 54 subjects tested positive. We report the expected number of subjects that could be tested using the 7,967 tests under the different strategies. For this measure, the perfect information lower bound on the expected tests per subject provides a *perfect information upper bound* (*UB*) on the expected number of subjects tested. Fig 5(A) displays the expected number of low-risk subjects that could be tested via 7,967 tests for the individual testing and robust pooling (n*) strategies, and the perfect information upper bound (*UB*), for a test sensitivity range [0.70, 1.00]. For each pooling strategy, the number of low-risk subjects screened reduces as test sensitivity increases. For example, as test sensitivity increases from 0.70 to 1.00, the number of low-risk subjects screened reduces from 66,010 to 54,898 under perfect information, and from 60,140 to 49,822 under robust pooling. This follows because a higher test sensitivity implies a higher likelihood that a pool containing an infected specimen will test positive, necessitating further individual testing for all subjects in the pool. As a result, optimal pool sizes are non-increasing in test sensitivity (Fig 1).

The sensitivity of the test may not be known with certainty. To examine the effect of the test sensitivity estimate, we calculate the robust pool sizes for the low-risk group for the week spanning April-2 to April-8, using three assumed test sensitivity values, of 0.70, 0.85, and 1. Fig 5(B) depicts the expected number of low-risk subjects that would be screened with 7,967 tests under a range of true test sensitivity values, *Se*, in [0.70, 1]. At the extremes, when the true sensitivity is 0.70 and the pooling strategy is based on a sensitivity of 1, 837.2 fewer subjects can be tested in expectation compared to using the true sensitivity; and when the true sensitivity is 1 and the pooling strategy is based on a sensitivity of 0.70, 540.4 fewer subjects can be tested in expectation.

Next, we study the effect of pooling on the expected number of *FN*s for various test sensitivity values. While pooling increases the number of *FN*s over individual testing for the same number of subjects (Eqs (5) and (6)), it also allows for expanded testing, thus reducing potential *missed cases* over individual testing (i.e., infected subjects not tested by individual testing, who could have been tested under pooling). To illustrate this point, we consider again the low-risk subjects tested during the week spanning April-2 to April-8 in the Iceland dataset. Fig 5(C) displays the number of *FN*s for both robust pooling and individual testing (for the 7,967 subjects tested under both strategies), as a function of test sensitivity, while Fig 5(D) displays the *FN*s for robust pooling, and the sum of *FN*s and missed cases for individual testing, for 52,173 subjects that would have been screened under robust pooling (with 7,967 tests).

## Discussion

In this paper, we provide a robust pooled testing approach to screen for COVID-19 and demonstrate its value for overcoming difficulties associated with COVID-19 testing. These difficulties include dynamic disease prevalence, which leads to high uncertainty in current prevalence, a wide range of possible test sensitivity values, different risk groups, and limited testing resources. For illustrative purposes, we use the Iceland dataset [7], in which the risk groups are based on whether the subject was tested by NUHI (high-risk due to symptoms and/or potential contacts) or by deCODE (low-risk, voluntary testing). The proposed robust pooling strategy significantly reduces the expected number of tests required to accomplish the screening conducted in Iceland for COVID-19 compared to individual testing, and the differences are more pronounced for the low-risk group, see Fig 3. Overall, the reductions in the number of tests are 54.2% to 61.9% for the high-risk group, for test sensitivity of 0.98 and 0.71, respectively, and 88.5% to 90.2% for the low-risk group, for test sensitivity of 0.98 and 0.71, respectively. These reductions are based on weekly forecasted point estimates and confidence intervals for the prevalence. The robust pooling strategy based on these forecasts does nearly as well as having perfect prevalence information (for which the respective reductions are 55.6% to 62.7% and 89.5% to 91.0%, for the high-risk and low-risk groups, for test sensitivity of 0.98 and 0.71), which, of course, we only have in hindsight. Thus, the proposed robust pooling strategy can be used to substantially expand COVID-19 testing capacity. This expansion is more pronounced at lower prevalence rates (due to fewer follow-up tests and larger pool sizes).

Dilution is an important issue when considering pooling, especially for large pools. In our models, we use a maximum allowable pool size, *n*_*max*_, to limit the dilution effect, which is a viable practice in pooled testing, especially when lab-based data on the magnitude of the dilution effect for different pool sizes is scarce (as is the case with COVID-19). Informed by the research that describes pool sizes for which pooling does not significantly affect the test sensitivity for the PCR test for COVID-19 (see Introduction), we consider that *n*_*max*_ is 32. Our analysis (Table 2) indicates that reducing *n*_*max*_, and thus further reducing the potential for dilution, does decrease the efficiency of robust poolng, but even at low values of *n*_*max*_, e.g., *n*_*max*_ = 5, robust pooling is still much more efficient than individual testing. As an alternative, if comprehensive data on the dilution effect for COVID-19 become available, one can derive a pooled sensitivity function, as a function of pool size, and use this function in the analytical expressions (Eqs (1), (3) and (5)) to model dilution (e.g., see [21, 34]). This will be an important future research direction once such data become available.

We demonstrate the benefits of pooling in more detail using the week of April-2 to April-8 of 2020 for the low-risk group (with 7,967 low-risk subjects individually tested) considering a test sensitivity of 0.85. In contrast to the 7,967 tests required for individual testing, robust pooling uses a pool size of 14, thus requiring $\left\lceil \frac{7,967}{14}\right\rceil =570$ pools. Given the true prevalence of 0.68% (calculated from the dataset), the probability that any pool will test positive (thus requiring individual follow-up tests for the subjects in the pool) is given by *Se*(1−(1−*p*)^*n*^) = 0.07722 (see Table 1). Therefore, for the 570 pools, we have, on average, 0.07722×570×14 = 616.2 individual follow-up tests. Thus, with pooling, the 7,967 subjects would require 1,186.2 (= 570+616.2) tests in expectation, an 85.1% reduction over individual testing. This reduction is closely related to a cumulative reduction in testing time, which we illustrate using the same week assuming a PCR testing machine that has a capacity of 96 tests, that is, 96 tests can run at the same time [25], and assuming that a test run takes 2 hours. Under individual testing, the number of machine runs is $\left\lceil \frac{7,967}{96}\right\rceil =83$, thus requiring 166 hours to complete testing. For the pooled strategy, $\left\lceil \frac{570}{96}\right\rceil =6$ runs are required for the pools, plus $\left\lceil \frac{616}{96}\right\rceil =7$ runs for the expected individual follow-up tests, thus requiring 26 hours of testing in expectation, compared to 166 hours for individual testing.

Next we discuss the effect of test sensitivity on pooling by examining Fig 5, which again considers the week of April-2 to April-8 of 2020 for the low-risk group of 7,967 subjects. If the 7,967 tests were used in the pooled strategy, between 49,822 (at a sensitivity of 1.00) and 60,140 (at a sensitivity of 0.70) subjects could have been tested (in expectation), see Fig 5(A). This is a considerable difference, and as Fig 5(B) depicts, even if the pooling strategy is derived under the wrong test sensitivity, pooling still does well, e.g., at a true sensitivity of 0.70, a strategy derived based on perfect sensitivity (of 1.00) tests 59,122 subjects, equivalently, 89.6% of those that could be tested if the true test sensitivity were known (i.e., derived using a test sensitivity of 0.70). This reduction is due to using pools that are too small (13 versus 15 under the true sensitivity of 0.70), which results in loss of efficiency, but this is somewhat mitigated by a lower probability that subjects in a pool will need individual follow-up testing. Fig 5(B) suggests that a good strategy to handle uncertainty in test sensitivity is to pick the mid-point of the potential sensitivity range.

Next, we compare the *FN*s under the different strategies. *FN* rate is larger under pooling than individual testing (Eqs (5) and (6)). For instance, Fig 5(C) shows that for a test sensitivity of 0.70, individual testing has 16 expected *FN*s, while pooling has 28 (for the 7,976 subjects). Of course, pooling uses many fewer tests for these 7,976 subjects, thus allowing for expanded testing. Considering this expanded testing population, Fig 5(D) shows that at a test sensitivity of 0.70, there are 171 *FN*s from pooling, and 16 *FN*s plus 335 missed cases from individual testing. As the test sensitivity increases, the *FN*s decrease fairly fast compared to the reduction in missed cases.

As we demonstrate in this study, robust pooling can substantially expand the testing capacity, and allow the testing of many more subjects for COVID-19. However, to achieve the maximum benefits of pooled testing, it is important to use pool sizes that are customized for the different risk groups in the population, and pool sizes that are designed to hedge against prevalence that is dynamic and uncertain; these are the key features of the robust pooling approach developed in this paper.

### Limitation

One limitation is the complex nature of the test sensitivity function for the PCR test for COVID-19. PCR tests tend to have high sensitivity, but for COVID-19, the clinical sensitivity may be lower [35] (due, for example, to low quality specimens), and the reasons for this lower clinical sensitivity, and ways for improving the clinical sensitivity, have implications for pooling.
